# Food Insecurity in Aging Communities: The Impact of Neighborhood Age Composition and Disadvantage on Food Access

**DOI:** 10.1093/geroni/igaf122.1241

**Published:** 2025-12-31

**Authors:** Samuel Van Vleet, Madeline Webber, Bailee Brekke, Erreannau Zellous

**Affiliations:** Miami University, Oxford, Ohio, United States; Miami University, Oxford, Ohio, United States; Miami University, Waddington, New York, United States; Miami University, Oxford, Ohio, United States

## Abstract

Food insecurity among older adults is a growing public health concern, yet little research has examined how the proportion of older residents in a neighborhood relates to food resource availability. This study investigates whether census tracts with higher proportions of people have access to food resources and explores how neighborhood disadvantage impacts this relationship. Using nationally representative data from the National Neighborhood Data Archive (NaNDA), we assess food insecurity risk across varying levels of neighborhood age composition, neighborhood disadvantage and other confounding factors. Findings indicate that census tracts with higher concentrations of older adults have reduced access to food resources, and this disparity is exacerbated in areas with greater neighborhood disadvantage. Furthermore, the interaction between neighborhood disadvantage and age composition suggests that older adult-dense areas experience compounded food insecurity when disadvantage is high. These results highlight the critical role of place-based disparities in shaping food access for aging populations. This study has significant implications for policymakers, researchers, and practitioners focused on addressing nutritional deficits in older adults. By identifying geographic disparities in food access, this study highlights the need for targeted interventions and policy solutions that consider both neighborhood age composition and disadvantage. Future research should explore community-driven strategies to improve food security and reduce disparities in disadvantaged aging communities.

